# Gray matter volume drives the brain age gap in schizophrenia: a SHAP study

**DOI:** 10.1038/s41537-022-00330-z

**Published:** 2023-01-09

**Authors:** Pedro L. Ballester, Jee Su Suh, Natalie C. W. Ho, Liangbing Liang, Stefanie Hassel, Stephen C. Strother, Stephen R. Arnott, Luciano Minuzzi, Roberto B. Sassi, Raymond W. Lam, Roumen Milev, Daniel J. Müller, Valerie H. Taylor, Sidney H. Kennedy, James P. Reilly, Lena Palaniyappan, Katharine Dunlop, Benicio N. Frey

**Affiliations:** 1 Neuroscience Graduate Program, McMaster University, Hamilton, ON Canada; 2 Faculty of Arts & Science, University of Toronto, Toronto, ON Canada; 3 Keenan Research Centre for Biomedical Science, Unity Health Toronto, Toronto, Canada; 4 Graduate Program in Neuroscience, Western University, London, ON Canada; 5 Robarts Research Institute, Western University, London, ON Canada; 6 Department of Psychiatry, Cumming School of Medicine, University of Calgary, Calgary, AB Canada; 7 Rotman Research Institute, Baycrest, Toronto, ON Canada; 8 Institute of Medical Science, University of Toronto, Toronto, ON Canada; 9 Department of Medical Biophysics, University of Toronto, Toronto, ON Canada; 10 Mood Disorders Program, Department of Psychiatry and Behavioural Neurosciences, Hamilton, ON Canada; 11 Women’s Health Concerns Clinic, St. Joseph’s Healthcare Hamilton, Hamilton, ON Canada; 12 Department of Psychiatry, University of British Columbia, Vancouver, BC Canada; 13 Departments of Psychiatry and Psychology, Queen’s University, and Providence Care, Kingston, ON Canada; 14 Department of Psychiatry, University of Toronto, Toronto, ON Canada; 15 Campbell Family Mental Health Research Institute, Centre for Addiction and Mental Health, Toronto, ON Canada; 16 Centre for Mental Health, University Health Network, Toronto, ON Canada; 17 Krembil Research Institute, University Health Network, Toronto, ON Canada; 18 Centre for Depression and Suicide Studies, and Li Ka Shing Knowledge Institute, St. Michael’s Hospital, Toronto, ON Canada; 19 Department of Electrical and Computer Engineering, McMaster University, Hamilton, ON Canada; 20 Department of Medical Biophysics, Western University, London, ON Canada; 21 Lawson Health Research Institute, London, ON Canada; 22 Department of Psychiatry, Western University, London, ON Canada; 23 Department of Psychiatry, Douglas Mental Health University Institute, McGill, Douglas, QC Canada; 24 Centre for Depression & Suicide Studies, Unity Health Toronto, Toronto, ON Canada; 25 Department of Psychiatry and Behavioural Neurosciences, McMaster University, Hamilton, ON Canada

**Keywords:** Biomarkers, Schizophrenia

## Abstract

Neuroimaging-based brain age is a biomarker that is generated by machine learning (ML) predictions. The brain age gap (BAG) is typically defined as the difference between the predicted brain age and chronological age. Studies have consistently reported a positive BAG in individuals with schizophrenia (SCZ). However, there is little understanding of which specific factors drive the ML-based brain age predictions, leading to limited biological interpretations of the BAG. We gathered data from three publicly available databases - COBRE, MCIC, and UCLA - and an additional dataset (TOPSY) of early-stage schizophrenia (82.5% untreated first-episode sample) and calculated brain age with pre-trained gradient-boosted trees. Then, we applied SHapley Additive Explanations (SHAP) to identify which brain features influence brain age predictions. We investigated the interaction between the SHAP score for each feature and group as a function of the BAG. These analyses identified *total gray matter volume* (group × SHAP interaction term *β* = 1.71 [0.53; 3.23]; *p*_corr_ < 0.03) as the feature that influences the BAG observed in SCZ among the brain features that are most predictive of brain age. Other brain features also presented differences in SHAP values between SCZ and HC, but they were not significantly associated with the BAG. We compared the findings with a non-psychotic depression dataset (CAN-BIND), where the interaction was not significant. This study has important implications for the understanding of brain age prediction models and the BAG in SCZ and, potentially, in other psychiatric disorders.

## Introduction

Schizophrenia (SCZ) is a major psychiatric disorder characterized by psychotic episodes, marked alterations in cognition, and impaired functioning with high rates of disability^1^. Brain imaging studies have shown that schizophrenia presents a chronic course accompanied by progressive brain alterations, such as gray and white matter volume loss and ventricle enlargement^2–4^. Some of these alterations have been postulated to be associated with a process named “accelerated aging”^5,6^. This hypothesis has led to a growing number of studies evaluating brain patterns suggestive of accelerated aging in psychotic and other major mental disorders^6–9^. These analyses involve the training of machine learning (ML) regression models to generate brain age predictions. The ML models are trained with structural neuroimaging data from healthy individuals paired with their chronological age. The underlying assumption is that the brain age of healthy individuals should match their chronological age. It was then demonstrated that these models overestimate brain age in individuals with SCZ (i.e., their brain age, based on ML model predictions, is higher than their chronological age)^8,10–13^. This difference between predicted brain age and chronological age is called the brain age gap (BAG). Meta-analyses found that BAG is correlated with chronological age in SCZ^14^, and longitudinal findings have demonstrated that the BAG increases in the first few years after illness onset^6^.

At first glance, these findings support the hypothesis of the neurobiological theory of accelerated aging in SCZ^5^. However, the brain features responsible for the BAG in SCZ have not been thoroughly investigated yet. A recent longitudinal study found an association between BAG changes and total gray matter volume reduction^15^. However, there is a gap in the understanding of model behavior, instead of reliance only on model final predictions. Studies focused on model analyses have solely investigated model-level explanations by extracting feature importance metrics from ML models, summarizing the contribution of all training data^16,17^. These findings have identified cortical thickness, ventricle volume, and subcortical volume of the thalamus and the putamen as the most relevant features for brain age prediction. The feature relevance analyses published thus far carry two limitations: (1) model-level feature importance precludes any investigation of whether or how certain features differentially contribute to individual model predictions; and (2) the most relevant features for the prediction of brain age in healthy subjects or individuals with SCZ may not be the same features that are associated with the BAG in SCZ. *Participant-level* explanations may help circumvent both limitations. SHapley Additive exPlanations^18^ (SHAP) is a participant-level explanation method that measures the marginal contribution of each feature to a given prediction generated by a tree-based nonlinear model. In other words, SHAP values are obtained for all features for each individual participant, resulting in a data matrix of the same dimensions as the original dataset of imaging features. Therefore, SHAP values can: (1) enable a group-level comparison of specific brain features that contribute most to age prediction; and (2) enable the estimation of the influence of individual features on each participant’s brain age prediction, thus allowing for a characterization of the relationship between BAG and SHAP values. As previous studies have found associations between BAG and total gray matter volume reduction, and model interpretations led to distinct drivers of brain age predictions, SHAP can help bridging this gap and improve the understanding of model interpretation.

Given the consistent findings of larger BAG in SCZ but with little understanding of the behavior of the brain age prediction models underlying this finding, we used 3 publicly available SCZ datasets (The Center for Biomedical Research Excellence [COBRE], MIND Clinical Imaging Consortium [MCIC], UCLA Consortium for Neuropsychiatric Phenomics [UCLA]) and an additional dataset of young individuals in early stages of SCZ (Tracking Outcome in Psychosis [TOPSY]) to understand what drives the BAG using SHAP values. Our primary objective was to identify the features from the brain age model that strongly drive the higher BAG in SCZ. Our secondary objectives were: (1) to estimate the group differences in feature contributions (SHAP values) to the brain age prediction; (2) to understand if the observed BAG effects are specific to established SCZ, by comparing the findings to a non-psychotic depression dataset (Canadian Biomarker Network for Depression [CAN-BIND]); and (3) to replicate previous findings on the positive BAG observed in SCZ.

## Results

### Model errors and group differences in BAG

To replicate the previous finding that SCZ exhibits greater BAG relative to HC, we began by checking brain age prediction errors in HC and then proceeded with *t* tests on the predictions of the two groups. A full description of the prediction procedures can be found in the methods section.

**Fig. 1:**
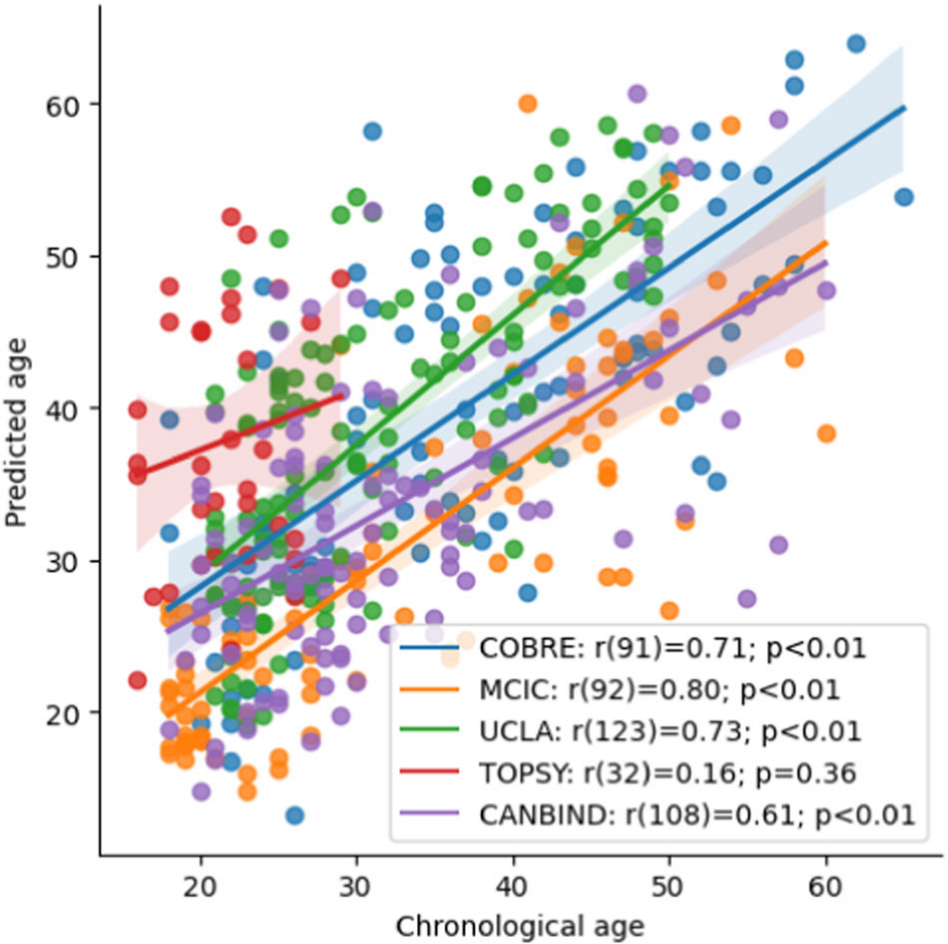
Association between predicted brain age and chronological age. Chronological age and brain age predictions for healthy comparison participants of each independent dataset.

The mean absolute errors of the brain age prediction model for HC in the COBRE, MCIC, UCLA, TOPSY, and CAN-BIND datasets were 7.21, 5.61, 8.02, 16.28, and 7.19 years, respectively. Their respective mean absolute percentage (MAPE) errors were 21.87%, 16.84%, 28.16%, 79.18%, and 23.52% with Pearson correlations of *r* = 0.71 (*p* < 0.01); *r* = 0.80 (*p* < 0.01); *r* = 0.73 (*p* < 0.01); *r* = 0.16 (*p* = 0.36); and *r* = 0.61 (*p* < 0.01) between predicted brain age and chronological age (Fig. 1).

The group difference in BAG was significant in two datasets, after adjusting for chronological age and sex. For COBRE, the group term was significantly associated with BAG (*β* = 4.80 95% CI [2.40; 7.20]; *p* < 0.01), with SCZ presenting a higher BAG than HC; chronological age was also significantly associated with BAG (*β* = −0.27 [−0.36; −0.17]; *p* < 0.01). The results were similar for the MCIC dataset: the SCZ group exhibited a larger BAG compared to HC (β = 6.87 [4.67; 9.04]; *p* < 0.01) and age was also significantly associated with the BAG (*β* = −0.11 [−0.20; −0.02], *p* = 0.02). In the UCLA dataset, the BAG was not associated with group (*β* = 2.10 [−0.40; 4.54]; *p* = 0.10), but it was significantly associated with both age (β = −0.15 [−0.27; −0.03]; *p* = 0.02) and sex (*β* = −3.67 [−5.87; −1.46]; *p* < 0.01). For TOPSY, BAG was not associated with group (β = 2.52 [−0.98; 6.20]; *p* = 0.16), sex, or age. In CAN-BIND, BAG was not associated with group (*β* = 0.69 [−1.20; 2.59]; *p* = 0.47), but it had a significant association with both age (*β* = −0.32 [−0.40; −0.25]; *p* < 0.01) and sex (*β* = −2.82 [−4.72; −0.92]; *p* < 0.01).

### Most relevant brain features for brain age prediction and group differences in SHAP values

**Fig. 2:**
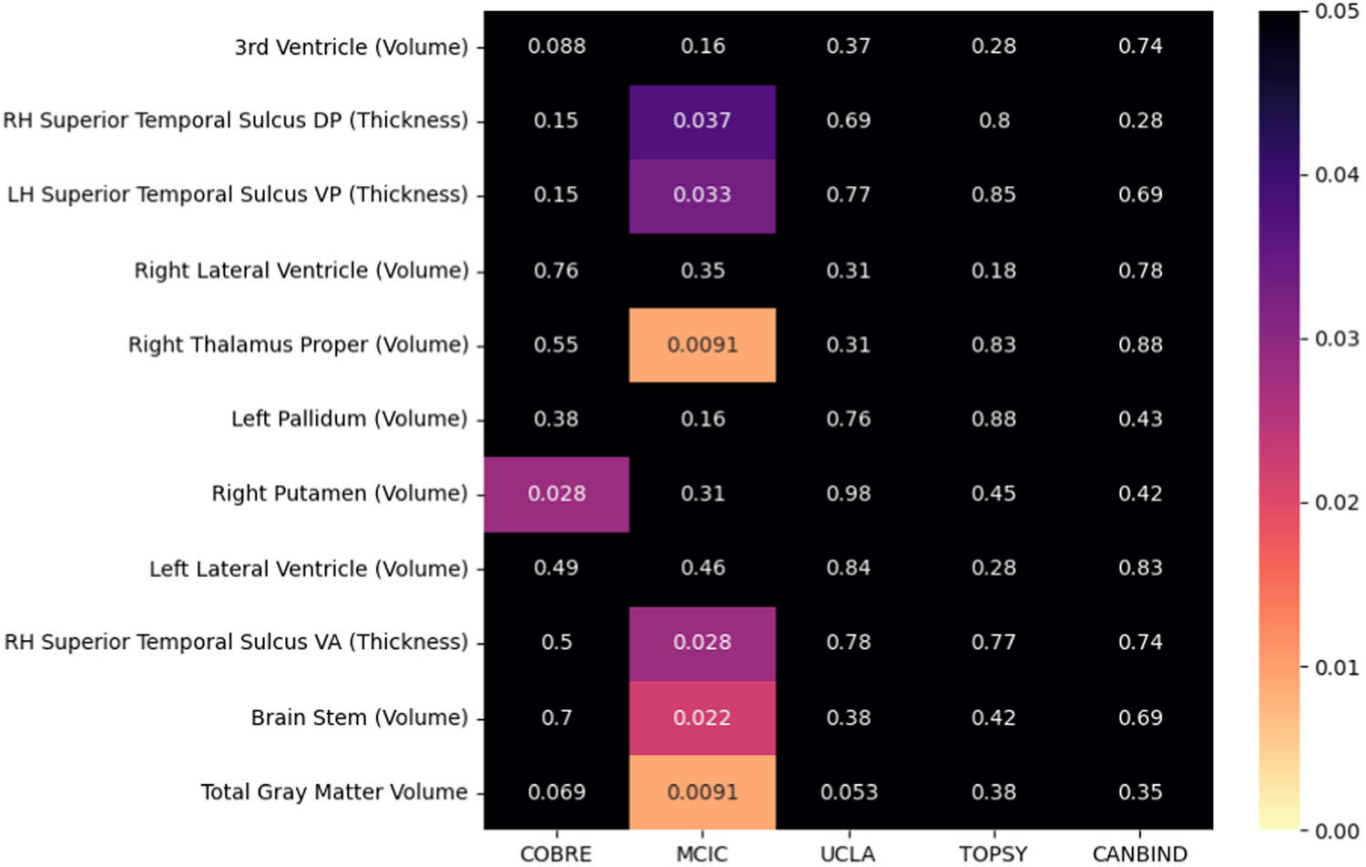
Group comparison of SHAP values between individuals with schizophrenia and healthy controls. *P* values for the difference in SHAP between SCZ and HC groups across datasets for the top 10 most relevant features of each source (11 in total, ranked in descending order of importance) after correcting for age and sex. *P* values are corrected by the false discovery rate method. DP dorsal posterior, VP ventral posterior, VA ventral anterior, RH right hemisphere, and LH left hemisphere.

To better understand which brain-based features contributed to BAG irrespective of diagnosis, we calculated the SHAP value for each of the 1,084 features and each participant and averaged this absolute value over all participants, regardless of group, in each dataset. The top ten most relevant features based on mean absolute contribution to the prediction were extracted from all five databases. We sought to analyze the top 10 most relevant features of each dataset, which represented a total of 11 unique features (high overlap of most relevant features across datasets). Mean SHAP values for these 11 features were compared between SCZ and HC using a Mann-Whitney U-test (Fig. 2). We adjusted for multiple comparisons with the false discovery rate method. For the COBRE dataset, the group difference in SHAP values was only significant for volume of the right putamen. For the MCIC dataset, the following features exhibited SHAP values that were significantly different between groups in: total gray matter volume, volumes of the brain stem and right thalamus, and thickness of both the right superior temporal sulcus (ventral anterior and dorsal posterior parts) and the left superior temporal sulcus (ventral posterior part). In the UCLA, TOPSY, and CAN-BIND datasets, none of the SHAP values were significantly different between groups.

### Group and SHAP interaction as a predictor of BAG

**Fig. 3:**
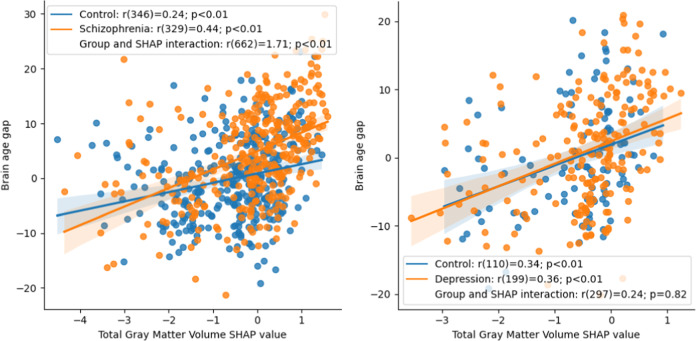
Assocation between brain age gap and total gray matter volume SHAP values. Univariate association between age-corrected brain age gap and total gray matter volume SHAP values for schizophrenia (left) and univariate association between age-corrected brain age gap and total gray matter volume SHAP values for depression (right).

**Fig. 4:**
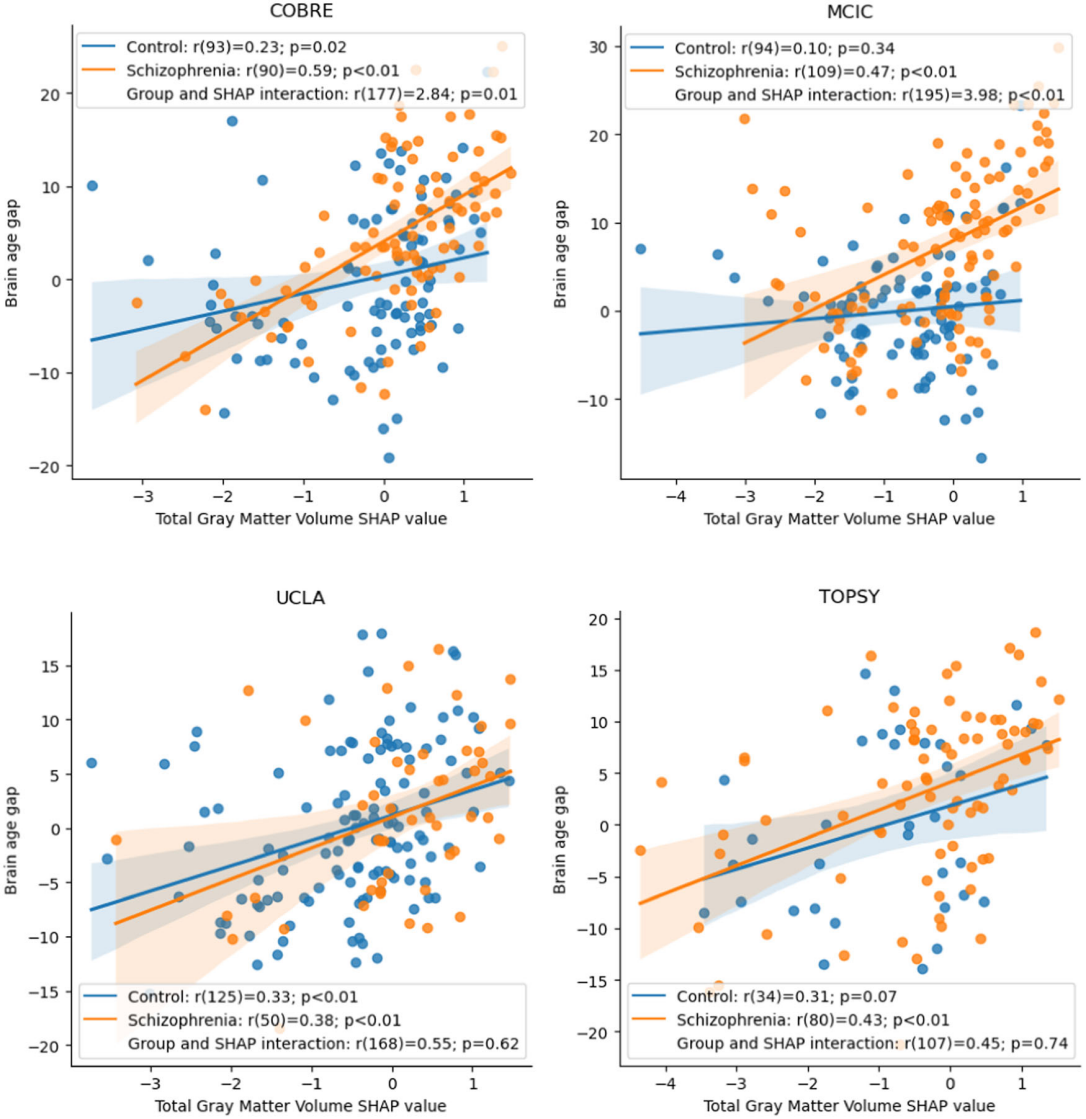
Association between brain age gap and total gray matter volume SHAP values for each individual database. Effects of the association between Total Gray Matter Volume SHAP and brain age gap (BAG) for each dataset of schizophrenia.

All four SCZ databases were combined to test the interaction between the mean SHAP values of each feature and group with respect to the BAG. This analysis identified brain features whose contributions to brain age prediction were more strongly associated with the BAG in one group more than in the other. For each of the 11 top brain features identified in the previous section, we tested a model of the relationship between BAG and the group × SHAP interaction, with group, SHAP, age, sex, and scanner as independent variables. In this analysis, only one group × SHAP interaction was significant: total gray matter volume (*β* = 1.71 [0.53; 3.23]; *p*_corr_ < 0.03). Using the database source instead of scanner effects as an independent variable did not change the significance of the model, with a marginal difference of 0.04 and 0.02 in the coefficients of total gray matter volume. The univariate relationship between BAG and SHAP values of total gray matter volume is displayed in Fig. 3. In Fig. 3, we also demonstrate that the group × SHAP interaction effect was not significant in depression (*β* = 0.24 [−1.86; 2.36]; *p*_corr_ = 0.83). This univariate relationship is demonstrated for each individual dataset of SCZ in Fig. 4.

## Discussion

The findings from this study shed light on the brain features underlying the consistent, yet mechanistically poorly understood, finding of a positive BAG in SCZ. Using SHAP analyses, we found that total gray matter volume was the strongest features influencing the BAG in SCZ. The thickness of the bilateral superior temporal sulcus and the volumes of certain subcortical structures—brain stem, putamen, and thalamus—also differed in SHAP values between SCZ and HC, but these respective interactions did not seem to be associated with the BAG. These findings have important implications in the way we interpret the BAG in SCZ and, possibly, other psychiatric disorders.

The strongest and only significant interaction signal in the BAG analyses came from the SHAP for total gray matter volume. This result is aligned with a large body of research describing gray matter reduction in SCZ^19–22^. Total gray matter volume reduction has been widely reproduced in SCZ research^2^ as well as being associated with the natural process of aging^23^. Recent work has also identified the longitudinal association between gray matter reduction and changes in BAG^15^. Other interactions with features representing smaller portions of the brain might exist that were not significant in our analysis due to the limitations imposed by the resolution of the MRI data and/or a stringent correction for multiple comparisons. Based on current pipelines of brain age prediction in SCZ, the BAG is mostly a reflection of the alterations observed in these two brain features. Moreover, our analysis revealed at least two other characteristics of the relationship between the BAG and brain age predictions. First, the feature importance order for brain age prediction (ranks from Fig. 2) does not determine the most relevant features for BAG group differences. For instance, the top 3 variables for brain age prediction (third ventricle volume, right hemisphere dorsal posterior superior temporal sulcus, and left hemisphere ventral posterior superior temporal sulcus) had no significant interaction with group when regressed on the BAG, meaning they did not influence the BAG in SCZ more than in HC. Secondly, group differences in SHAP values of certain features (*p* values from Fig. 2) do not reflect the influence of those features on the BAG observed in SCZ.

Notably, the association between BAG and total gray matter volume SHAP were distinct in the two investigated disorders, with a significant association in SCZ but not in depression. This finding is in line with previous reports suggesting that the accelerated aging effect in depression is not present to the same extent as in SCZ^14^. Moreover, this finding also suggests that despite some similarity in magnitude and spatial distribution, the pathophysiological basis of grey matter reduction in MDD likely differs from SCZ.

Findings varied across databases, particularly in age prediction performance and SHAP value differences between groups. These differences can be largely attributed to the differences in age and sex distributions. For instance, TOPSY has a much younger group, which is known to heavily affect brain age predictions, which tend to be overestimated in younger individuals and underestimated in older individuals^24^. This effect was corrected for the downstream analyses, but it is unlikely to have been eliminated. Additionally, a younger group would present less of the effects of the disorder due to reduced illness duration, treatment exposure, and number of episodes, indicating that BAG is a process that is more pronounced after the first episode, and thus may reflect a dynamic adaptive process as argued elsewhere^25^. Importantly, the interaction between total gray matter volume SHAP and BAG was only significant in two of the four datasets (COBRE and MCIC), as shown in Fig. 4, but the direction of the association is the same across datasets.

Our findings support previous studies showing significant alterations in total gray matter volume in SCZ^26^. It also supports research suggesting that the BAG changes are mostly associated with total gray matter volume reduction^15^. A limitation of previous studies, however, was that linear associations between BAG and imaging variables were not able to determine whether the BAG effects were driven by the changes in gray matter or were simply correlated. In the current study, we have demonstrated that these features, when considered in a multivariate setting and their estimated marginal contributions, are the ones that explain the BAG in SCZ. In other words, BAG likely represents an individualized, age-adjusted overall gray matter volume reduction in SCZ. Furthermore, it is important to note that clinical interpretations should not rely on variable importance for the prediction of brain age, as we demonstrated that the top variables for brain age prediction are not the same as the top variables for the BAG in SCZ.

Beyond providing an explanation for the BAG in SCZ, our analyses have also contributed to the understanding of the behavior of brain age prediction models in SCZ. Our first analysis replicated previous findings of BAG in SCZ. Most previous studies, with a few exceptions in epigenetic analyses^27,28^, have consistently demonstrated patterns suggesting “accelerated aging” in SCZ^6–8,10,11^. Although the UCLA dataset did not present a significant group difference in BAG, this dataset has the smallest sample size and an age-matching issue. Our secondary analysis presents a novel finding that the SHAP values of certain brain features differ significantly between HC and SCZ, which indicates that the ML model behaves differently between the groups. Overall, these results suggest that specific structural brain characteristics of individuals with SCZ affect the behavior of the model when predicting brain age in those individuals. The discrepancy of findings across databases may be attributed to the heterogeneity within and between cohorts and/or differences in MRI acquisition across datasets.

Our study should be interpreted in light of some limitations. First, algorithms other than XGBoost might exhibit different behavior than what was uncovered in this study, although recent studies have demonstrated that different model types lead to similar results in brain age and feature importance^29^. Specific characteristics of the training sets used in different studies may lead to different outcomes. Also, the training dataset from Kaufmann et al.^12^ may have included some of the samples from the COBRE and MCIC datasets. We analyzed the potential for overfitting and only a small subset of participants had a prediction error of less than one year. This result indicates that our findings are unlikely to be affected by this issue. Finally, this study was based on structural brain features, which may not capture the association of brain age/BAG with functional brain features as assessed with functional MRI or electroencephalography. This study also presents several strengths. The use of public datasets and publicly available models allows this study to be fully reproducible and its findings may be independently verified. Additionally, to the best of our knowledge, the use of SHAP for comparing groups is a relatively novel approach in brain age research. Irrespective of the technical specifics of how the BAG is defined and how the model behaves, BAG has been shown to be an important imaging marker for the disorder^30,31^. From predicting mortality risk^32^ to predicting risk of dementia^33^, there are many potential applications of the BAG in health-related research.

Future studies should consider exploring a wider set of ML models and their different behaviors in predicting brain age. Feature importance methods other than SHAP may also be useful to further investigate model behavior, such as LIME^34^ and counterfactual generation^35^. Other datasets and analyses in SCZ with more in-depth clinical characterization may also help to identify the effects of medication use, lifestyle, quality of life, BMI, smoking status, and other measures that may impact findings. A natural next step is to investigate model behaviour in other neurological diseases and psychiatric disorders, such as bipolar disorder and Alzheimer’s disease, both of which have been reported to exhibit a BAG^36,37^.

In conclusion, this study has demonstrated the potential of feature explanation methods to clarify what contributes to brain age prediction and the BAG in schizophrenia. The brain-age gap in schizophrenia is driven mainly by *total gray matter volume*, despite grey matter volume itself being a feature less predictive of an individual’s age than other brain features. These findings may open new venues to improve the interpretation of BAG findings in SCZ and other psychiatric disorders.

## Methods

### Databases

Five databases were included in this study: a study from The Center for Biomedical Research Excellence (COBRE)^38^, the MCIC database^39^, the UCLA Consortium for Phenomics database^40,41^ (UCLA), the Tracking Outcome in Psychosis (TOPSY) dataset, and the Canadian Biomarker Network for Depression (CAN-BIND) dataset. The demographic characteristics of the participants from each independent database that were included in this study are presented in Table 1.

**Table 1:** Demographic characteristics of participants from included databases.

Database		Controls	Cases	Total	*p* value
COBRE	*N*	93	90	183	
	*Age*				0.99
	Mean (SD)	37.63 (11.66)	37.61 (13.66)	37.62 (12.65)	
	Range	18–65	18–65	18–65	
	*Sex*				0.28
	Female	26 (27.96%)	18 (20.00%)	44 (24.04%)	
	Male	67 (72.04%)	72 (80.00%)	139 (75.96%)	
MCIC	*N*	109	94	203	
	*Age*				0.47
	Mean (SD)	32.64 (11.97)	33.81 (11.20)	33.27 (11.55)	
	Range	18–60	18–60	18–60	
	*Sex*				0.26
	Female	30 (31.91%)	26 (23.85%)	56 (27.59%)	
	Male	64 (68.09%)	83 (76.15%)	147 (72.41%)	
UCLA	*N*	125	50	175	
	*Age*				<0.01
	Mean (SD)	31.53 (8.80)	36.46 (8.88)	32.94 (9.07)	
	Range	21–50	22–49	21–50	
	*Sex*				0.01
	Female	59 (47.20%)	12 (24.00%)	71 (40.57%)	
	Male	66 (52.80%)	38 (76.00%)	104 (59.43%)	
TOPSY	*N*	34	80	114	
	*Age*				0.03
	Mean (SD)	21.47 (3.40)	23.75 (5.79)	23.07 (5.28)	
	Range	16–29	16–49	16–49	
	*Sex*				0.31
	Female	10 (29.41%)	15 (18.75%)	25 (21.93%)	
	Male	24 (70.59%)	65 (81.25%)	89 (78.07%)	
CANBIND	*N*	110	199	309	
	*Age*				0.15
	Mean (SD)	32.88 (10.70)	34.94 (12.63)	34.21 (12.00)	
	Range	18–60	18–61	18–61	
	*Sex*				1.00
	Female	70 (63.64%)	128 (64.32%)	198 (64.08%)	
	Male	40 (36.36%)	71 (35.68%)	111 (35.92%)	

The COBRE dataset includes HC participants (*N* = 93) and participants diagnosed with SCZ (*N* = 90). A multi-echo MPRAGE sequence was used to collect neuroimaging data from all participants alongside basic sociodemographic and clinical information. The database also includes functional neuroimaging data that was not used in this study.

The MCIC is a database of participants in the early course of their illnesses and includes HC participants (*N* = 109) and participants diagnosed with SCZ/schizoaffective disorder (*N* = 94) The MCIC database also includes functional and diffusion-weighted imaging data that were not used in this study. Data used in the preparation of this work were obtained from the Mind Clinical Imaging Consortium database through the Mind Research Network (www.mrn.org). The MCIC project was supported by the Department of Energy under Award Number DE-FG02-08ER64581. MCIC is the result of efforts of co-investigators from University of Iowa, University of Minnesota, University of New Mexico, Massachusetts General Hospital, where participants were recruited.

The UCLA database includes HC participants (*N* = 125) and participants diagnosed with SCZ (*N* = 50). The database includes extensive neuropsychiatric and cognitive assessments alongside anatomical and functional neuroimaging data. Participants were recruited through the community and local clinics. A 3T Siemens Trio scanner was used to collect the data. Functional and diffusion-weighted imaging data are also available and were not used in this study. This data was obtained from the OpenfMRI database (https://openfmri.org/dataset/ds000030/). Its accession number is ds000030.

TOPSY is a high-resolution 7T MRI study of early-stage schizophrenia with an untreated patient group and patients with established treatment for >3 years (Clinical Trials Identifier: NCT02882204). T1 structural imaging data were acquired using a Siemens MAGNETOM 7.0T MRI (Erlangen, Germany) [8-channel transmit/32-channel receive, head-only, radiofrequency coil] at the Centre for Metabolic Mapping at Western University in London, Ontario. We included 34 healthy controls and 80 patients (*n* = 66 with first-episode schizophrenia of which *n* = 28 were antipsychotic naive (35% of all patients), and *n* = 36 were exposed to antipsychotics but had not received minimal effective dose for 2-weeks or more (47.5% of all patients); *n* = 14 with established schizophrenia with treatment exposure for 3 years minimum (17.5% of all patients). Inclusion criteria for first-episode patients included (1) having <2 weeks of lifetime exposure to antipsychotic medications, and (2) being at their first clinical presentation of psychotic symptoms. More details on this sample can be found in Liang et al.^42^.

The Canadian Biomarker Integration Network in Depression (CAN-BIND) is major depressive disorder clinical trial dataset where individuals received escitalopram treatment (10–20 mg/d) for 8 weeks and a subsequent 8 weeks of escitalopram or adjunctive therapy of escitalopram and aripiprazole for non-responders of escitalopram monotherapy. Structural imaging data from baseline, when study participants were unmedicated, was used to calculate brain age. The dataset is comprised of 6 centers across Canada^43^.

### Brain age prediction and age correction

The brain age prediction models (one for males and one for females) were pretrained in a large database in another study^12^. The authors of this study made their models available online (https://github.com/tobias-kaufmann/brainage). The code was executed in R (version 3.6.3). In line with the original study, we used separate models for males and females. The models are gradient-boosted trees generated by the XGBoost^44^ method. The models rely on 1084 features from the Human Connectome Project (HCP) atlas^45^. There are 360 features for volume, 360 for surface area, and 360 for cortical thickness (180 from each hemisphere), alongside 30 subcortical volumes and 8 summary variables. The average chronological ages from the pretrained models were 48.01 for males and 46.63 for females.

Brain age predictions suffer from an age dependency, for which an age-correction procedure was conducted^24^. This procedure fits a linear model between predicted age and age of HC participants. Then, an age-corrected predicted age is derived for both HC and SCZ based on the slope, intercept, and predicted age extracted from the HC group. The rationale for this method is to ensure that the model has a consistent error across the lifespan for HC participants. This procedure was done independently for each database. All further analyses, except for group comparisons of the BAG to avoid circular analysis, used the age-corrected BAG.

### Image preprocessing

All five databases underwent the same pipeline of feature extraction with slight adjustments in their processing steps. Images that were available in DICOM format were converted into NIFTI format using the *dcm2niix and dcm2nii* tools. For the COBRE dataset, which had multi-echo scans, the root mean squared equivalent volume was used. Then, the *recon-all* command from FreeSurfer (version 6.0.0 and version 7.2.0 for TOPSY) was run for each scan. We used the multimodal HCP^41^ atlas to extract the features that are expected by the brain age prediction model, spanning volume, area, and thickness measures. The segmentations were checked for major registration and out-of-the-brain segmentation errors using the platform VisualQC (https://github.com/raamana/visualqc) (0.5.2).

### Deriving participant-level explanations using SHAP

Unlike linear regression models, where each feature is associated with a coefficient that may be interpreted as its average contribution across samples, nonlinear models are not as simple. In the case of nonlinear tree-based models, each feature may have a different contribution depending on the path an individual prediction took in the tree. Therefore, model-level interpretations based on average feature contribution do not fully explain each prediction. To circumvent this issue, model explanations need to be derived at a participant level, detailing how each independent prediction was derived. SHAP is a method based on game theory that extracts marginal contributions of features from predictions^46^. The SHAP value for a specific feature for an individual prediction may be understood as the difference in the prediction when that feature is left out of the decision tree (marginal contribution). To illustrate, in a database with N participants and M features, SHAP generates a table of N × M explanations, where each value V^(i,j)^ represents the contribution of feature *j* to the model prediction of participant *i*. SHAP values were extracted using the SHAPforxgboost package in R (https://cran.r-project.org/web/packages/SHAPforxgboost/index.html). In the case of XGBoost, SHAP values represent the contribution of each feature to the deviation of the prediction from the mean age of the database (the starting point of the XGBoost model).

### Statistical analyses

All statistical analyses were performed in Python (version 3.7.4) with Scipy (version 1.3.1) and Statsmodels (version 0.10.1). The statistical analyses are separated into three parts: (1) replication of previous findings of BAG differences between SCZ and HC, (2) group comparison of SHAP values for each of the 11 most important features, and (3) BAG as a function of the interaction between group and SHAP and other covariates.

First, we analyzed whether there were significant differences in the BAG between HC and SCZ participants separately for each database. This difference was assessed using a general linear model, with BAG as the dependent variable and group as the independent variable, with age and sex included as covariates. To avoid circular analysis, this part was done with the BAG prior to age-correction. Subsequent analyses were conducted with age-corrected values.

Second, we investigated whether the SHAP values were different between groups separately for each database. We limited the comparisons to the union of the 10 most relevant features based on their mean absolute contribution across groups from the five databases. We employed a Mann-Whitney U-test due to the non-normal distribution of SHAP values.

Finally, we combined datasets to assess whether there was a differential effect of feature contribution to the BAG between groups using an interaction term. This modeling was done using robust regression to avoid the shortcomings of linear models^47^. The age-corrected BAG was the dependent variable, while age, sex, group, SHAP, and group × SHAP were the independent variables. The group × SHAP term was of interest, as it represents the difference between groups in how SHAP values relate to the BAG.

## Data Availability

All code to reproduce the experiments are available on GitHub (https://github.com/Ballester/brain-age-shap). The public datasets are accessible through their respective platforms. For access to TOPSY and CAN-BIND, please contact the corresponding authors.
